# Capillary-like Formations of Endothelial Cells in Defined Patterns Generated by Laser Bioprinting

**DOI:** 10.3390/mi12121538

**Published:** 2021-12-10

**Authors:** Lothar Koch, Andrea Deiwick, Boris Chichkov

**Affiliations:** 1 Institut für Quantenoptik, Leibniz Universität Hannover, Welfengarten 1, 30167 Hannover, Germany; deiwick@iqo.uni-hannover.de; 2 Niedersächsisches Zentrum für Biomedizintechnik, Implantatforschung und -Entwicklung, Stadtfelddamm 34, 30625 Hannover, Germany

**Keywords:** bioprinting, laser, capillaries, vascularization, endothelial cells, tissue engineering, biofabrication

## Abstract

Bioprinting is seen as a promising technique for tissue engineering, with hopes of one day being able to produce whole organs. However, thick tissue requires a functional vascular network, which naturally contains vessels of various sizes, down to capillaries of ~10 µm in diameter, often spaced less than 200 µm apart. If such thick tissues are to be printed, the vasculature would likely need to be printed at the same time, including the capillaries. While there are many approaches in tissue engineering to produce larger vessels in a defined manner, the small capillaries usually arise only in random patterns by sprouting from the larger vessels or from randomly distributed endothelial cells. Here, we investigated whether the small capillaries could also be printed in predefined patterns. For this purpose, we used a laser-based bioprinting technique that allows for the combination of high resolution and high cell density. Our aim was to achieve the formation of closed tubular structures with lumina by laser-printed endothelial cells along the printed patterns on a surface and in bioprinted tissue. This study shows that such capillaries are directly printable; however, persistence of the printed tubular structures was achieved only in tissue with external stimulation by other cell types.

## 1. Introduction

Three-dimensional tissues produced from human cells are a promising substitute for animal testing, and it is hoped that one day it will be possible to produce entire replacement organs for transplantation. However, this is still a long way off. Many artificially produced tissues are still quite simple, containing only a few cell types. Bioprinting is one of the technologies that has been increasingly used in recent years, and has the potential to produce complex tissue structures [1,2]. Bioprinters print a bioink (biological material with embedded cells) and deposit it layer by layer as drops or strands. In some cases, the term bioprinting is defined much more broadly [3], but here we will focus on technologies that are used to print cells.

Usually, nozzle-based techniques—such as extrusion printers [1,2,4,5,6,7,8] or inkjet printers [9,10]—are used for this purpose. Thereby, shear forces are generated in the nozzle that can destroy the cells in the bioink [10,11]. To prevent this, a compromise must be chosen between the nozzle diameter and the associated print resolution on the one hand, and the viscosity of the bioink and the cell density in the bioink on the other. Thus, one can either use a small-diameter nozzle (e.g., 100 µm [8]) and achieve high resolution with thin ink and low cell density, or use a large nozzle (e.g., 0.5 to 1 mm diameter [6]) and be relatively free in the choice of bioinks and cell density at low resolution.

To overcome this limitation, a laser-based nozzle-free printing technology is used by some groups, including ours, which allows for the printing of cells with a combination of high resolution (a few tens of micrometers), high-viscosity bioink (1 mPa s to 1 Pa s), and high cell density (up to 10^8^ per mL) [12,13]. In this process, the energy of laser pulses is used to create small vapor bubbles at the interface between a solid substrate—usually a glass slide—and a thin layer of bioink. These vapor bubbles expand and re-collapse within microseconds, thereby expelling small amounts of the bioink as a small jet perpendicular to the surface [14]. The bioink jets then deposit as droplets on a second substrate to be printed on, e.g., a second glass slide or a scaffold. When printing cells, droplet sizes in the nano- to picoliter range can be achieved [15]; without cells, even smaller droplets are possible.

Up to a certain tissue thickness—which is usually a few hundred micrometers, depending on the tissue type and cell density—the cells can be supplied by diffusion if the tissue piece is immersed in a nutrient solution, e.g., in a cell culture medium [16]. In order to be able to produce larger tissue pieces, a vascular network is essential for supplying the cells, i.e., 3D tubular vascular structures through which the nutrient solution can be pumped, which is called perfusion [17].

The natural vascular system in the human body consists of vessels of very different diameters, from ~3 cm (the aorta) to less than 10 µm in capillaries [18]. Capillaries consist of a single layer of endothelial cells (ECs) and a basement membrane with isolated pericytes. Reconstructing complex vascular systems with all different vessels by means of tissue engineering is very challenging, and has not yet been successfully demonstrated [18,19,20,21]. Generally, there are different approaches to creating blood vessels embedded in tissue [17,20,22,23], differing in terms of whether well-defined or more random blood vessel patterns are created, what size of blood vessels can be created, how soon the blood vessels can be perfused, and to what extent one is still free in the design of the rest of the tissue. There are many approaches in which larger vessels are made in a defined way—as microfluidic systems [24], or even by bioprinting [4,5,6,25,26,27]—and the capillaries then develop by sprouting in random patterns [28] or from randomly distributed ECs [24,29,30]. However, for some applications it might be necessary to create a defined network of capillaries, since some natural organs and tissues have highly structured vascular systems, e.g., the liver [31] or kidneys [32]. In addition, directly generated capillary networks could more quickly lead to cells being adequately supplied, which would prevent necrosis.

One approach for creating perfusable vascular networks is to apply a placeholder material placed at the site of these vessels, e.g., nylon threads [30] or a sacrificial material that is subsequently dissolved by water [4], temperature [29,33], or enzymes [34]. The surrounding tissue is usually formed by casting a gel with cells over the threads or sacrificial material structure. After removal of the placeholder material, cell culture medium containing endothelial cells is injected in the tubular cavities. The endothelial cells adhere and line the cavities from the inside. This is a promising approach, allowing the cells to be supplied in a timely manner; however, many tissues also contain other complex structures, so casting methods will often be insufficient.

If bioprinting processes are already used to produce tissues, it would therefore be desirable to also produce the entire vascular system, including the capillaries, together with the rest of the tissue, layer by layer, in a defined pattern. Whether it is possible to ‘directly’ print endothelial cells in such a way that they form closed tubes with diameters as small as those of capillaries in natural tissue along the printed patterns is the subject of the present study. High-resolution bioprinting such as laser-based bioprinting, in principle, offers the possibility of printing tissue with integrated capillaries. However, this involves the printing of dissociated ECs, which would then have to form into vessels with a lumen. These cells do not do this automatically; they require certain biochemical stimuli, such as growth factors, including the vascular endothelial growth factor (VEGF). These growth factors are expressed in tissue by other, undersupplied cells to stimulate vessel growth and the sprouting of new vessels in their vicinity, known as angiogenesis.

To date, there are only a few publications reporting studies on the direct printing of small capillary-like structures from ECs; most of them also applied laser-based bioprinting. Wu et al. [35] printed human umbilical vein endothelial cells (HUVECs) and human umbilical vein smooth muscle cells (HUVSMCs) on Matrigel, but at a much lower cell density of 10^6^–10^7^ cells per mL, as opposed to the approximately 10^8^ cells per mL we used here; it is unclear whether vessels were actually formed in this work [35], as this was not demonstrated; their printed patterns look more like single elongated cells in many places.

Gaebel et al. [36] printed HUVECs and mesenchymal stem cells from human bone marrow onto a patch made of polyester urethane urea and immersed in Matrigel, which they applied to a myocardial infarction zone in rats; it was shown that the printed vascular cells were integrated into the rat vasculature; however, it cannot be concluded from this that vascular structures would also have been formed ex vivo. Gruene et al. [37] imprinted endothelial colony-forming cells and adipose-derived stem cells into a fibrin matrix; they observed a distinct interaction between the cells and the joint formation of shorter strand-like structures; whether a lumen was formed within them has not been investigated. Kérourédan et al. [38] printed strips of endothelial cells several hundred micrometers wide, within which randomly distributed capillary-like structures were then formed, but whether these structures had a lumen has not been demonstrated. Yanez et al. [6] printed human dermal microvascular endothelial cells in fibrin between layers of collagen with human dermal fibroblasts and epidermal keratinocytes, and implanted these skin constructs into full-thickness wounds in mice; they used an inkjet printer for this purpose, in contrast to the other publications mentioned above; they were able to show accelerated wound healing compared to commercial wound dressings without ECs, along with the formation of blood vessels in the skin constructs within two weeks; however, the development of vascular structures ex vivo has not been investigated.

In the study described here, we investigated the conditions that must be met during laser printing of dissociated endothelial cells in order to enable them to form capillary structures with lumina ex vivo. For this purpose, we applied two approaches: First, we printed endothelial cells in lines and grid patterns on a layer of Matrigel. Matrigel is derived from Engelbreth–Holm–Swarm mouse sarcoma, and is known to be very rich in various growth factors, including VEGF [39]; it is widely used in 2D and 3D cell culture, including bioprinting [35,36]; however, its composition is rather undefined.

We varied the droplet size and, thus, the number of cells per unit of length. We investigated whether these cells formed a continuous tubular structure, whether all cells were involved in this process, and what diameters of tubes could be produced. In the second approach, we printed tissue from skin cells, fibroblasts, and keratinocytes, with an integrated grid pattern of endothelial cells. In this case, the stimuli should come from the other cells—especially from the fibroblasts. The aim was to create continuous tubular microcapillary structures with small diameters in the order of 10 µm. In contrast to the structures on the Matrigel layer, these microcapillaries could not be viewed directly with a microscope, so histological sections of the skin pieces were prepared.

## 2. Materials and Methods

### 2.1. Laser-Based Bioprinting

**Figure 1:**
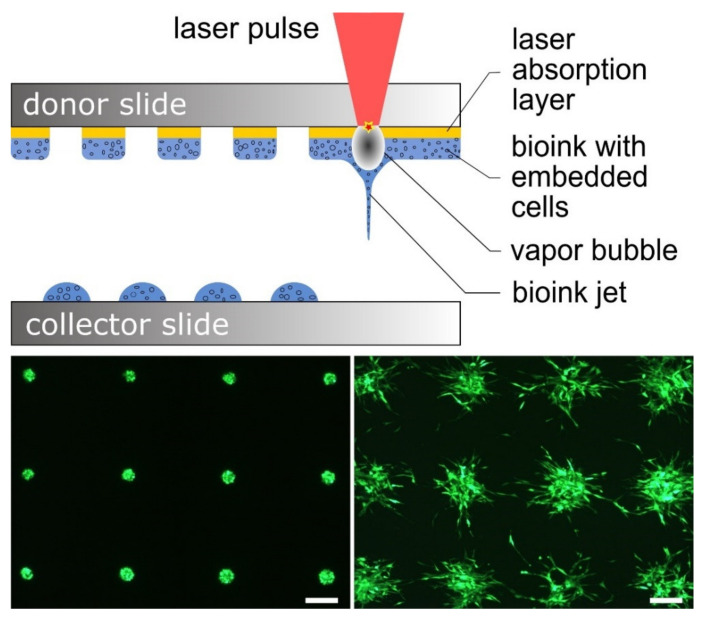
The laser bioprinting technique. Top: Schematic representation of laser-based bioprinting; in the focus, the laser pulses vaporize material of an absorption layer located between a glass plate and the bioink with dissociated cells. The vapor pressure temporarily creates a vapor bubble that expels the underlying bioink as a jet. The bioink settles as drops on a collector slide or other object to be printed onto. Bottom: Microscopic images of fluorescent cells (NIH/3T3 murine fibroblasts, GFP-labeled, printed on Matrigel; distance between droplets is 600 µm) immediately after printing (**left**) and after two days (**right**); the behavior of the cells after printing depends on the cell type and many other factors, and must be considered in a printing strategy. The fibroblasts depicted here behave very differently on Matrigel compared to the endothelial cells used in this study. Scale bars are 200 µm.

For printing of cells, a laser-based printing process was used that has already been described in previous publications [14,15,40,41,42,43,44]. This process is based on the so-called laser-induced forward transfer, and is shown schematically in Figure 1. In principle, the setup consists of a pulsed laser, a transparent substrate for the laser radiation—in our case, a glass slide that we call the donor slide—and a second substrate onto which the cells are printed; this can also be a glass slide, and is called the collector slide here.

The donor slide is first thinly coated with a material that absorbs the laser radiation; in our experiments, this was a 60 nm thin gold layer applied by sputter coating. On top of this absorption layer, a layer of bioink with the suspended cells is coated; in the experiments described here, the thickness of this layer was ~45 to 60 µm, depending on the bioink used.

The donor slide is then mounted upside down in the printing setup. The laser pulses are focused from above through the glass into the absorption layer. This layer is vaporized at the focus, creating an expanding vapor bubble that expels the bioink underneath. The vapor bubble collapses after only a few microseconds, but due to the inertia of the bioink it continues to move and forms a jet that lasts for a few hundred microseconds and then breaks off [14]. In this process, the bioink with the suspended cells is deposited as a droplet on the collector slide. The droplet size can be varied from a few picoliters to a few nanoliters [15] by adjusting the laser pulse energy, bioink viscosity, and layer thickness. By moving the laser focus and the donor slide with respect to the collector slide, any 2D pattern can be printed, and a 3D construct can be created layer by layer. The collector slide can be coated before printing, or different microfluidic structures or scaffolds can be positioned on it; cells can be printed onto these coatings or into these structures.

In our experiments, a Nd:YAG laser (DIVA II; Thales Laser, Orsay, France) with 1064 nm wavelength, approximately 10 ns pulse duration (FWHM), and 20 Hz repetition rate was applied. The laser pulses were focused with a 50 mm achromatic lens into a 40 µm diameter ablation spot. Depending on the bioink and intended droplet size, the laser pulse energy was set between 6 and 13 µJ, corresponding to laser fluences around 0.5–1 J/cm^2^. The distance between donor slide and collector slide or the upper layer of the printed skin tissue was adjusted to 1 mm.

Previous studies have demonstrated that no heat shock is induced to the printed cells [42], and that the printing process itself does not significantly reduce cell viability, while applied biomaterials, bioink, or collector coating material may have substantial and cell-type-specific effects on viability [43]. With cell culture medium used as a bioink, high survival rates close to 100% have already been demonstrated in a previous study [44]. With the collagen used here, we also observed very high viability, but this was not quantified in printed 3D tissue.

### 2.2. Cell Culture

Neonatal normal human epidermal keratinocytes (NHEKs) and human umbilical vein endothelial cells (HUVECs) were obtained from Lonza (Basel, Switzerland). The NHEKs were cultured under serum-free conditions in keratinocyte growth medium (KGM-Gold, Lonza) supplemented with bovine pituitary extract, insulin, recombinant human epidermal growth factor (hEGF), transferrin, hydrocortisone, epinephrine, amphotericin, and gentamicin in cell culture flasks (TPP-Techno Plastic Products AG, Trasadingen, Switzerland) coated with rat tail collagen type I (5 µg/cm^2^, Fisher Scientific, Schwerte, Germany). The cells were maintained in a humidified incubator with 5% CO_2_ at 37 °C, and the medium was changed three times per week. For all experiments, NHEKs of passages 2–4 were used. The HUVECs were cultured in cell culture flasks coated with rat tail collagen type I (5 µg/cm^2^) in the endothelial cell growth medium-2 BulletKit (EGM-2, Lonza), consisting of EBM-2 basal medium supplemented with 2% fetal bovine serum (FBS), hEGF, vascular endothelial growth factor, human recombinant fibroblast growth factor basic, ascorbic acid, hydrocortisone, human recombinant insulin-like growth factor, heparin, amphotericin, and gentamycin. The cells were maintained in a humidified incubator with 5% CO_2_ at 37 °C, and the medium was changed twice per week. For all experiments, HUVECs of passages 3–5 were used. Human foreskin HFF-1 fibroblasts (DSZM, Braunschweig, Germany) were cultured in Dulbecco’s modified Eagle’s medium F-12 (DMEM/F12) supplemented with 10% FBS (Biochrom, Berlin, Germany) and 1% penicillin/streptomycin (Biochrom), with two medium changes per week. The human pulmonary microvascular endothelial cell line (HPMEC-ST1.6R, immortalized) was kindly provided by C. James Kirkpatrick [45,46]. The cells were maintained in EGM-2 medium in a humidified incubator with 5% CO2 at 37 °C, and the medium was changed twice per week. For some experiments, the HPMEC-ST1.6R endothelial cells were labeled for green fluorescent protein (eGFP) expression by stable transduction with the lentiviral vector (pRRL.PPT.SF.FP.pre) [40].

### 2.3. Printing Experiments

Two different printing experiments were performed. In the first set of experiments, HPMECs were printed on Matrigel. The printing parameters were adjusted to achieve very high cell density at high resolution, i.e., small droplet size. For this purpose, HPMECs were suspended in EGM-2 medium as bioink, at a concentration of 100 million cells per mL, and applied as a 45 µm thick layer on the gold-coated donor glass slide. The collector glass slides were coated with a 100 µm thick layer of a mixture of two-thirds Corning™ Matrigel™ and one-third EGM-2 medium. The laser pulse energy was set to between 6 and ~10 µJ, depending on the intended droplet size. The printed patterns consisted of grids or lines in which the droplets were printed close to one another so that there were no spaces without cells within a line, resulting in a distance between neighboring droplets of 40 µm. After printing, the samples were kept in an incubator (37 °C, 5% CO_2_) for half an hour before they were covered with EGM-2 medium and placed back in the incubator. The samples were analyzed directly after printing and after 24 and 48 h by fluorescence microscopy using an Axio Imager A1.m microscope (Carl Zeiss, Oberkochen, Germany) equipped with an AxioCam ICc1 camera and AxioVision Rel. 4.8 software. Due to calibration with a reference object, this software enabled the measurement of the size of objects in images.

In the second set of experiments, pieces of skin were printed with an integrated grid of endothelial cells. The printing of skin was analogous to our previous publications [40,41] using collagen as bioink, but with different skin cells. A collagen solution of Corning rat tail collagen type I (3.8 mg/mL: 74% of the total volume) was prepared with sodium hydroxide (NaOH 0.25 mol/L, 6% of the total volume) for neutralization and gelation, 10x PBS (10% of the total volume), and DMEM/F12 medium (10% of the total volume) and kept on ice to avoid premature gelation. The gelation of the collagen bioink occurred soon after the printing of each layer at room temperature, allowing us to print 3D structures. A piece of MatriDerm™ (Dr. Suwelack Skin & Health Care, Billerbeck, Germany)—a 1 mm thick spongy collagen–elastin matrix—soaked with the collagen solution and placed on the collector glass slide was used as the base of the skin pieces for providing mechanical stability. On top of this matrix, 9 layers of HFF-1 fibroblasts were printed first, followed by a grid pattern of HUVECs, then another 9 layers of HFF-1 fibroblasts, and finally 9 layers of NHEK keratinocytes. Here, the concentration of cells in the collagen solution as bioink was 20 million per mL, and a 60 µm thick layer of the bioink was applied on the gold-coated donor glass slide. The laser pulse energy was set to 13 µJ for these printing experiments, and the distance between printed droplets was 80 µm. After printing, the skin samples were kept in an incubator (37 °C, 5% CO_2_) for half an hour before they were covered with DMEM/F12 medium and placed back in the incubator.

### 2.4. Immunostaining of 3D Cell Constructs

For immunofluorescence analysis, cell constructs were embedded in Tissue-Tek OCT (Science Services, München, Germany) and snap-frozen in liquid nitrogen. Frozen sections (7 µm) were fixed in 4% paraformaldehyde/PBS for 10 min and permeabilized with 0.3% Triton X-100/PBS. After blocking with 2% bovine serum albumin/PBS solution at 37 °C for 1 h, the sections were incubated with primary antibodies overnight at 4 °C. Applied antibodies were anti-CD31 (ab28364 rabbit polyclonal antibody; Abcam, Cambridge, UK) and anti-cytokeratin 14 (sc-58724 mouse monoclonal antibody, clone LL002; Santa Cruz Biotechnology, Heidelberg, Germany). After several washing steps, the sections were incubated with a fluorescence-conjugated secondary antibody at an appropriate dilution (Alexa Fluor^®^ 488 or Alexa 555 conjugated Donkey anti-rabbit/anti-mouse IgG (H + L); Fisher Scientific, Schwerte, Germany) for 1 h at 37 °C. Cell nuclei were stained with Hoechst 33,342. The samples were analyzed by fluorescence microscopy using the Axio Imager A1.m microscope.

## 3. Results

### 3.1. Printing of Endothelial Cells on Matrigel

In the first series of printing experiments, HPMECs were printed with cell culture medium as bioink onto a layer of Matrigel. Since the Matrigel, which was previously mixed with cell culture medium at a ratio of 2:1, did not have a high stiffness, the jet of bioink and cells always penetrated the Matrigel slightly, creating a slight depression (not shown) in which the drop of bioink remained.

**Figure 2:**
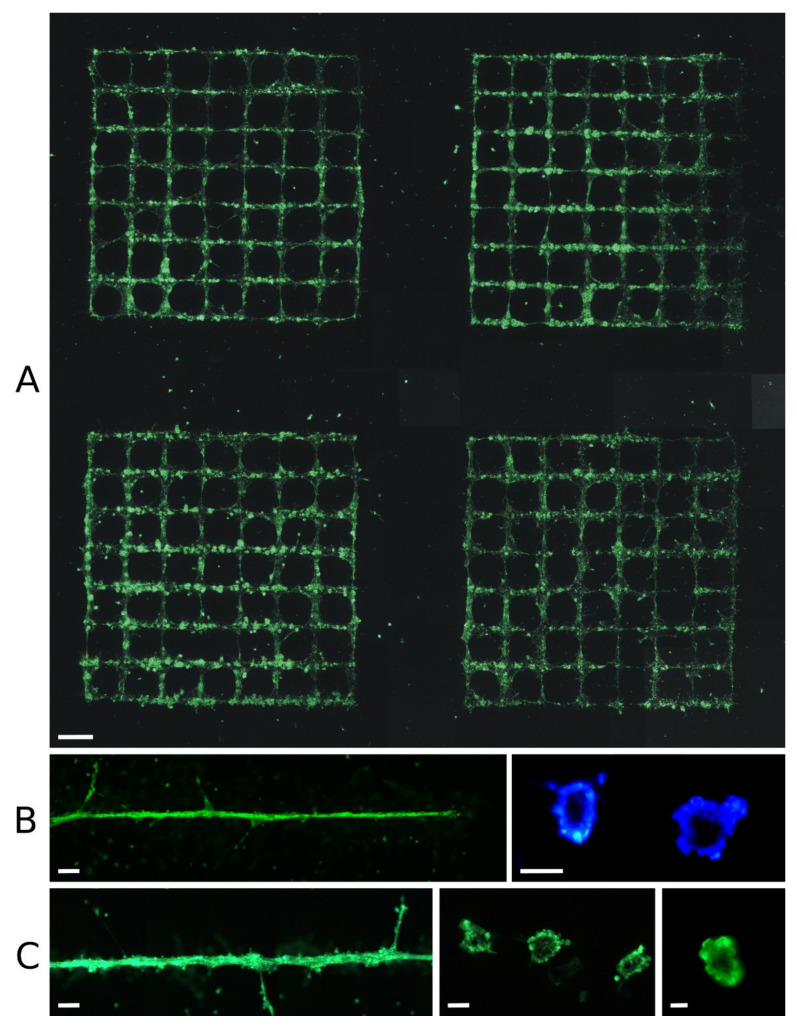
Vessel formation on Matrigel within 24 h. Green fluorescent endothelial cells (HPMECs, GFP-labeled) printed in patterns of grids (**A**) or as a line (**B**,**C**) on Matrigel. Each image was taken 24 h after printing. In rows B and C on the right-hand side there are cross-sections of printed HPMEC vascular structures, which were additionally stained with Hoechst 33,342 (**B**); the printed cells formed a tubular structure with a lumen. In principle, printing of more complex patterns (such as the grids shown in (**A**)) is possible; however, none of the grids is perfect—each has breaks in lines where cells have disappeared and other places where cells have formed larger agglomerates. Some cells have moved significantly away from their printed position. The applied laser pulse energy was 6 µJ, and the patterns in panels (**A**,**C**) on the left were printed twice in the same place. Scale bars are 1 mm (**A**), 200 µm (**B** left and **C** left), 50 µm (**B** right and **C** center), and 10 µm (**C** right).

When drops of cell culture medium containing endothelial cells were printed in close succession as a line on Matrigel, the cells often formed tubular structures within ~12 to 24 h. Figure 2 shows such structures printed in grids (row A) or lines (rows B, C). In rows B and C, tubular vascular structures formed from the printed cells after 24 h are shown; they were ~30 to 100 µm in diameter. Individual endothelial cells also appear to have formed even smaller sprouting substructures, although it is not clear whether these were previously part of the main tubular structure. At the bottom right, two cross-sections through printed vascular structures are shown, at ~15 to 50 µm in diameter. While nearly all cells became part of the tubular structure in B, there were additional cell clusters on the surface of the vessel structure in C.

Panel A shows a printed sample 24 h after printing, consisting of four grids, each with 7 mm side length and 1 mm line spacing. Although the overall grid structure is clearly visible in all four grids, none of the grids had become perfect after 24 h. Some of them contained imperfections with areas without cells along the printed lines. There were also some diagonal cross-connections that were not intended. In some cases, cells had formed larger spherical agglomerates, or there were areas where the cells remained scattered and spread over the surface of the Matrigel instead of staying together. Although there were of course variations in cell density and number of printed cells per unit length, the observed imperfections often occurred after printing, when the cells formed into clusters.

However, when endothelial cells are printed with medium, some cells are always non-adherent after half an hour, and can be washed away when cell culture medium is added. Therefore, after 24 h, some cells were always clearly visible outside the printed patterns.

**Figure 3:**
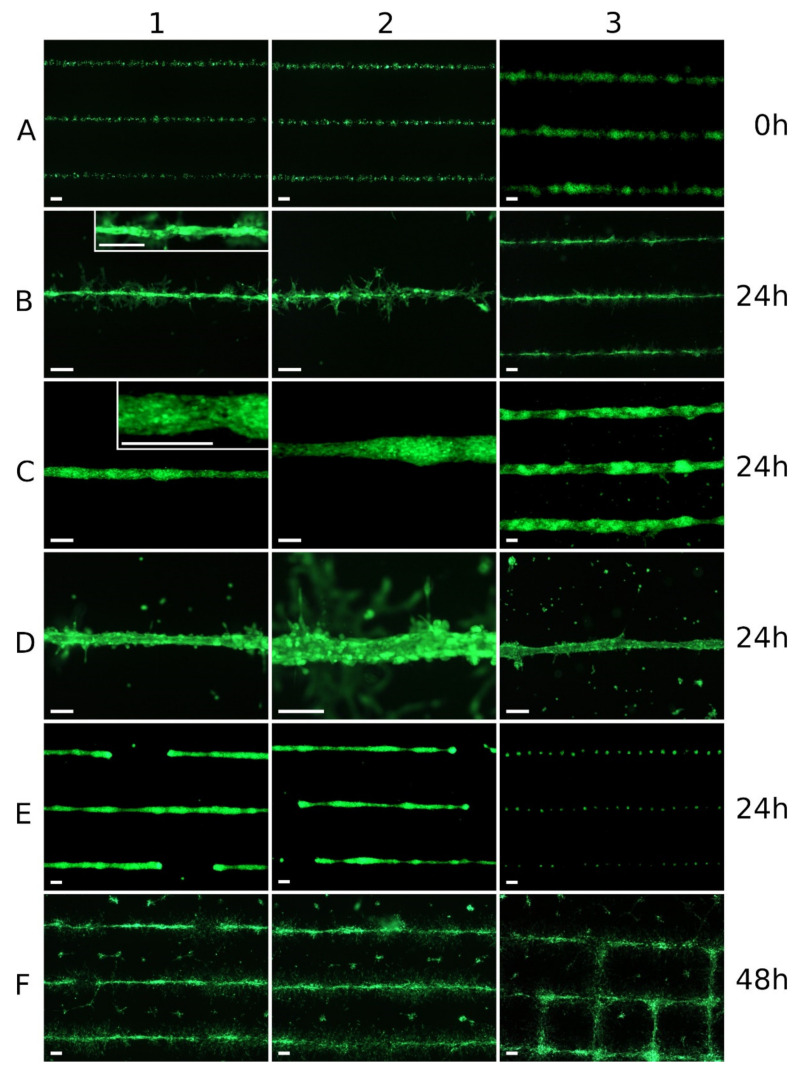
Vessel formation was not always successful. Green fluorescent endothelial cells (HPMECs, GFP-labeled) printed in thinner and thicker lines on Matrigel. Images were taken immediately (line **A**), 24 h (**B**–**E**), or 48 h (**F**) after printing. Despite very similar appearance of the printing results immediately after printing, the patterns may look very different after 24 h. Examples of thinner lines (line **B**) and thicker lines (lines **C** and **D**) are shown after 24 h, where continuous tubular structures have formed entirely (**B1**,**C1**,**C2**,**C3**) or partially (**B3**). In **B3**, the cells have formed partial aggregates but no vascular structures; in line **D** they are only gutter-shaped structures that are open at the top. In line **E**, after 24 h, larger cell-free interruptions can appear, although directly after printing there was a continuous line; in extreme cases, the endothelial cells contract to equidistant spherical cell aggregates (**E3**). The formation of the tubular structures was transient only; within another 1–2 days, endothelial cells began to re-dissolve the formation, migrate (**F**) and, also by proliferation, gradually colonize the entire area. Applied laser pulse energies were 6 µJ (**A1**,**A2**,**B**,**E3**,**F**), 8 µJ (**A3**,**C1**,**D**,**E1**,**E2**), and 10 µJ (**C2**,**C3**); the patterns in panels (**C**,**D**,**E1**,**E2**,**F**) were printed twice in the same place. **B1** and **C1** include an integrated detail image. All scale bars are 200 µm.

Figure 3 shows how very different cell structures developed from relatively similar printed patterns within 24 h. In row A, printed lines from smaller droplets (A1 and A2; approx. 60 µm droplet diameter) and from larger droplets (A3; approx. 120 µm droplet diameter) can be seen immediately after printing. In row B, printed lines from smaller droplets can be seen after 24 h. From the line in panel B1, a continuous tubular structure had emerged, and additional endothelial cells were located around this vascular structure and in contact with it. In contrast, no continuous tubular structure had emerged from the line in panel B2; there were several aggregates of cells, perhaps including a lumen, but also areas along the line with isolated cells. In the three lines in B3, there were fragmented tubular structures, but with many interruptions.

Rows C and D show printed lines of larger droplets after 24 h. While the cell formations along the lines in row C had a largely closed outer endothelial layer, there were many more cells inside with a spherical shape, which had not become part of the cell formation. The cells shown in row D did form continuous formations, but these formations were similar to gutters rather than tubes, i.e., open at the top. Inside these gutters there were further cells, which were not part of the formations. The individual cells and cell aggregates, which were clearly away from the lines, had been washed away when the cell culture medium was applied half an hour after printing, as they were not yet adherent at that time.

Row E shows cell formations that had also emerged from continuous printed lines within 24 h. Here, one can see (E1 and E2) larger areas without cells, because they had migrated from these areas to the cell formations. An extreme example can be seen in image E3. Here, the endothelial cells had contracted from a very homogeneous printed line into a series of almost equidistant spherical cell aggregates within 24 h.

In general, the tubular vascular structures formed after laser printing of endothelial cells were transient (F). On the one hand, the cells continued to proliferate, but the tubular cell aggregates also disintegrated in the following two days, and the endothelial cells gradually spread relatively evenly over the entire surface and overgrew it.

### 3.2. Printing of Skin Constructs with Embedded Endothelial Cells

In the second series of printing experiments, patterns from endothelial cells (HUVECs in this case) were integrated into a three-dimensional printed skin tissue. Rat tail collagen type I was used as a bioink for this purpose. A layered structure was printed with endothelial cells in a grid pattern as a layer between the 9th and the 10th of 18 layers of fibroblasts; finally, nine layers of keratinocytes were printed on top.

**Figure 4:**
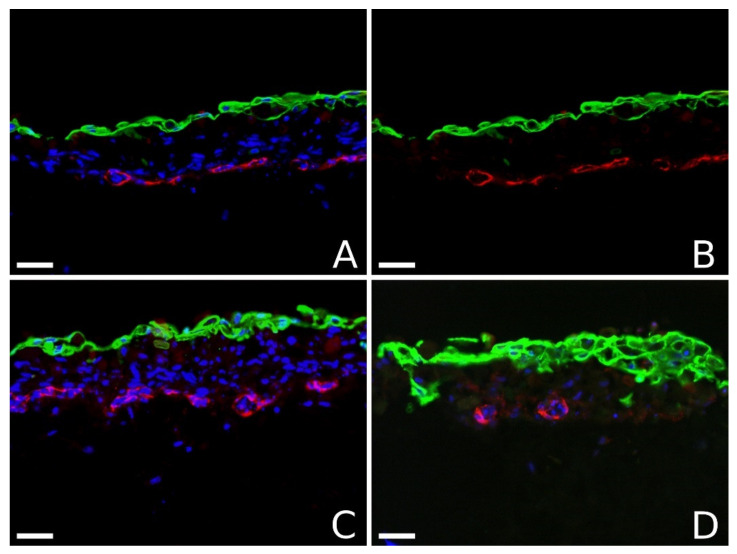
Vessel formation in bioprinted skin tissue. Sections through printed skin constructs consisting of human dermal fibroblasts with an integrated grid of human endothelial cells and human epidermal keratinocytes. Rat tail collagen type I was used as the bioink. Sections were prepared three days after printing, and staining was performed using cytokeratin 14 (green; stains keratinocytes), CD31 (red; stains endothelial cells), and Hoechst 33,342 (blue; stains all nuclei). Panels (**A**,**B**) show the same section depicted on the right without Hoechst 33,342 nuclear staining; they clearly show that the endothelial cells have formed a tubular structure, through which the section passes several times. Panel (**C**) shows a section that is partially vertical and partially oblique through capillaries. In panel (**D**), two vessels are sectioned vertically. Scale bars are 100 µm.

Figure 4 shows four vertical sections through these skin pieces. On these images, the endothelial cells were stained red with antibodies against CD31, while the keratinocytes were stained green with antibodies against cytokeratin 14. In addition, all cell nuclei were stained in blue with Hoechst 33,342. Separate staining for fibroblasts was not performed, because the appearance and location of endothelial cells was the primary focus of our study. The keratinocytes often formed only a rather thin stratification.

The images in Figure 4 clearly show the formation of tubular structures by the endothelial cells three days after printing. In panel B, the blue nucleus staining is removed, while in panel D it is attenuated to make the tubular structure more visible. In panel B, a tubular structure runs almost exactly along the cut edge, but meanders around it and is therefore cut multiple times, making the tubular structure particularly visible. In panel D, two vascular structures can be seen in cross-section.

## 4. Discussion

The behavior of cells after printing depends very much on the cell type, the presence of other cells, and the materials used: on the one hand, on the applied bioink; on the other hand, on the material onto which the cells were printed.

The different behavior of different cell types under similar conditions can be seen by comparing Figure 1 (bottom right) and Figure 2 and Figure 3. While fibroblasts (Figure 1) rapidly migrated apart after printing on Matrigel, and soon spread relatively evenly over the surface, ECs initially formed into cell aggregates, and later dissociated and migrated. We hypothesize that this later phase, when the EC aggregates dissolved again, resulted from the diffusion of growth factors and other chemical stimuli from the Matrigel into the medium, leading to a marked decrease in concentration and the disappearance of significant gradients of these agents. Furthermore, it could be that the Matrigel layer does not sufficiently replace all necessary stimuli in perfused tissue to maintain capillary structures for a longer period. We also observed such different behavior of different cells under similar conditions in a previous study [37], in which ECs and adipose-derived stem cells (ASCs) were printed in fibrin gel. In that study, in contrast to the ASCs, the endothelial cells initially did not migrate at all.

In the current study, the Matrigel strongly stimulated laser-printed endothelial cells, which is not surprising, since Matrigel is derived from cancer tissue; stimulation of the supplying blood vessels is essential for the growth of a cancerous tumor [47].

As a result, the endothelial cells printed on Matrigel formed tubular structures. When these cells were printed in such a way that they were present in a thin line at a very high cell concentration of ~100 million per mL, it was possible to ensure that all or almost all of these ECs formed together into a single tubular vascular structure with a lumen inside (Figure 2B); the diameter ranged from just over 10 µm to ~60 µm.

A cell density of 100 million cells per mL is the density found in epidermal tissue, for example; 100 million endothelial cells per mL in the bioink means that the cell pellet accounts for nearly one-third of the total volume. Significantly higher cell densities could no longer be printed, as the bioink would then consist only of cells. Lower endothelial cell densities were printed (not shown), but with a halved cell density the smallest capillaries already could not be generated. Therefore, only printing with 100 million cells per mL is presented here.

When the droplet size was increased so that more cells were present to form a vascular structure with a larger diameter, not all of the cells participated in the forming of this tubule. Instead, ECs remained inside in a spherical shape, or additional cell clusters formed on the surface (Figure 2C, left). With even larger and more (printing twice on the same position) droplets and, thus, potentially even larger vessel diameters, no continuous outer endothelial layer was formed at all, but there were isolated small and larger openings, or the tubular structure was no longer fully formed at all (Figure 3D).

In general, however, the structures could only ever be produced with limited controllability and reproducibility. In larger patterns, such as the grids in Figure 2A, there were always voids, variations in the diameter of the vascular structures, or formation of rather spherical cell aggregates—especially at crossing points. Despite many attempts, we did not succeed in printing a grid pattern that was a completely perfect grid of vascular vessels after 24 h. This was not due to the printing itself, but to the behavior of the cells in the following hours. In some cases, cells printed in a clean line contracted into cell aggregates, leaving behind completely cell-free areas up to 1 mm long. In extreme cases—perhaps in ~1–2% of the experiments—a series of equidistant spherical cell aggregates emerged from a uniform line of endothelial cells within less than 24 h.

The distances were usually all the same on one sample, but varied from sample to sample. The periodic variation is not seen immediately after printing. We assume that this is a biochemical effect between the cells; however, we do not know what it is or why it leads to the equidistance.

Regarding the success of the process in printing on Matrigel, it can be said that there was never a completely perfect larger structure after 24 h, and sometimes there were no vascular structures at all. In most cases, there were structures that looked like vessels from above, although how much of the total length of printed lines became vessel structures varied widely. There were areas where the structures clearly looked like vessels; we made histological sections of such structures, which always showed a lumen (Figure 2). However, there were also areas where the structures did not look quite as good, but could still contain lumina, so no quantitative statements can be made here about the percentage of successful lumen formation.

In addition, all vascular structures that formed within 12–24 h were transient, and dissolved after only two days. The proliferation of the cells probably also contributed to this behavior, but we assume that the main reason could be the decreasing stimulation by the bioactive substances in the Matrigel.

In contrast, a more permanent stimulation is provided by other cells that express growth factors themselves. This has already been shown in the study by Gruene et al. [37]. Here, we wanted to investigate whether printed lines of ECs surrounded three-dimensionally by other cells—as is common in tissue—would also form capillary structures. Therefore, we printed skin tissue in layers with an integrated layer of ECs in a grid pattern, as depicted in Figure 2A. As shown in Figure 4, we also observed the formation of vascular structures with lumina in this case. The diameter of well-formed vascular vessels was comparable to that on Matrigel, in the range of ~10 to ~50 µm. This size range coincides roughly with the size range of capillaries in natural tissue; vessels with larger diameters usually contain smooth muscle cells as well (arterioles and venules).

We saw vessels with lumina in all sections of the samples printed with the optimal parameters, but not always in all of them that we expected. Since the sections were not selected to be representative, no quantitative statement on the percentage of completed lumen formation can be made here. However, more than half of the capillaries were present with the lumina that would ideally be present.

In addition to direct printing of ECs, there are further methods of producing such small capillaries in defined patterns integrated into tissue. For example, Zheng et al. [33] recently published a study in which they showed that the principle of printing a sacrificial material as a placeholder for subsequent vessels also works for very small vessels with cross-sections of 60 or 30 µm. To do this, they used Pluronic F127 as the sacrificial material and a special electrohydrodynamic inkjet printing process that can print much smaller droplets (without cells) compared to normal inkjet printers. They then poured a gelatin methacryloyl gel with human dermal fibroblasts mixed in over the sacrificial material structure and allowed it to gel. After liquefying and removing the pluronic and applying an internal capillary coating with fibronectin, they injected HUVECs into the vessels and cultured the constructs for up to three weeks. The HUVECs uniformly lined the small vessels in the construct. These results are very promising, but do not yet allow conclusions to be drawn as to whether large pieces of tissue or entire organs could be produced using such methods.

In conclusion, our study demonstrates that laser-based bioprinting can be used to create the smallest capillary vessels in predefined patterns from endothelial cells without sacrificial materials. However, in order to be able to print thicker tissue, these capillary vessels must still be combined with larger vessels, and together they must then be perfusable. If this tissue construct with vessels is connected to tubing and a pump, this system would combine biological and non-biological vessels from 10 µm to ~1 mm—a range of three orders of magnitude. Studies have already been published in which other bioprinting techniques were used to create and perfuse defined functional vessels from less than 200 µm [4] to 1 mm in diameter [6], but these vessels did not yet have the full three-layer structure of natural vessel walls. It also remains to be seen whether it will be possible to create all vessel sizes using a single bioprinting technique, or whether this will require a combination of multiple approaches.

## References

[1] Li J., Chen M., Fan X., Zhou H. (2016). Recent advances in bioprinting techniques: Approaches, applications and future prospects. J. Transl. Med..

[2] Kačarević Ž.P., Rider P.M., Alkildani S., Retnasingh S., Smeets R., Jung O., Ivanišević Z., Barbeck M. (2018). An Introduction to 3D Bioprinting: Possibilities, Challenges and Future Aspects. Materials.

[3] Sasmal P., Datta P., Wu Y., Ozbolat I.T. (2018). 3D bioprinting for modelling vasculature. Microphysiol. Syst..

[4] Miller J., Stevens K.R., Yang M.T., Baker B., Nguyen D.-H.T., Cohen D., Toro E., Chen A.A., Galie P., Yu X. (2012). Rapid casting of patterned vascular networks for perfusable engineered three-dimensional tissues. Nat. Mater..

[5] Zhang Y., Yu Y., Chen H., Ozbolat I.T. (2013). Characterization of printable cellular micro-fluidic channels for tissue engineering. Biofabrication.

[6] Kolesky D.B., Homan K.A., Skylar-Scott M.A., Lewis J.A. (2016). Three-dimensional bioprinting of thick vascularized tissues. Proc. Natl. Acad. Sci. USA.

[7] Lee W., Lee V., Polio S., Keegan P., Lee J.-H., Fischer K., Park J.-K., Yoo S.-S. (2010). On-demand three-dimensional freeform fabrication of multi-layered hydrogel scaffold with fluidic channels. Biotechnol. Bioeng..

[8] Faulkner-Jones A., Greenhough S., King J., Gardner J., Courtney A., Shu W. (2013). Development of a valve-based cell printer for the formation of human embryonic stem cell spheroid aggregates. Biofabrication.

[9] Yanez M., Rincon J., Dones A., De Maria C., Gonzales R., Boland T. (2015). In Vivo Assessment of Printed Microvasculature in a Bilayer Skin Graft to Treat Full-Thickness Wounds. Tissue Eng. Part A.

[10] Cui X., Dean D., Ruggeri Z.M., Boland T. (2010). Cell damage evaluation of thermal inkjet printed Chinese hamster ovary cells. Biotechnol. Bioeng..

[11] Chang R., Nam J., Sun W. (2008). Effects of Dispensing Pressure and Nozzle Diameter on Cell Survival from Solid Freeform Fabrication–Based Direct Cell Writing. Tissue Eng. Part A.

[12] Lin Y., Huang Y., Chrisey D.B. (2009). Droplet formation in matrix-assisted pulsed-laser evaporation direct writing of glycerol-water solution. J. Appl. Phys..

[13] Guillotin B., Souquet A., Catros S., Duocastella M., Pippenger B., Bellance S., Bareille R., Rémy M., Bordenave L., Amédée J. (2010). Laser assisted bioprinting of engineered tissue with high cell density and microscale organization. Biomaterials.

[14] Unger C., Gruene M., Koch L., Koch J., Chichkov B. (2011). Time-resolved imaging of hydrogel printing via laser-induced forward transfer. Appl. Phys. A.

[15] Gruene M., Unger C., Koch L., Deiwick A., Chichkov B. (2011). Dispensing pico to nanolitre of a natural hydrogel by laser-assisted bioprinting. Biomed. Eng. Online.

[16] Radisic M., Malda J., Epping E., Geng W., Langer R., Vunjak-Novakovic G. (2006). Oxygen gradients correlate with cell density and cell viability in engineered cardiac tissue. Biotechnol. Bioeng..

[17] Yang G., Mahadik B., Choi J.Y., Fisher J.P. (2020). Vascularization in tissue engineering: Fundamentals and state-of-art. Prog. Biomed. Eng..

[18] Fleischer S., Tavakol D.N., Vunjak-Novakovic G. (2020). From Arteries to Capillaries: Approaches to Engineering Human Vasculature. Adv. Funct. Mater..

[19] Datta P., Ayan B., Ozbolat I.T. (2017). Bioprinting for vascular and vascularized tissue biofabrication. Acta Biomater..

[20] Richards D., Jia J., Yost M., Markwald R., Mei Y. (2017). 3D Bioprinting for Vascularized Tissue Fabrication. Ann. Biomed. Eng..

[21] Cao X., Maharjan S., Ashfaq R., Shin J., Zhang Y.S. (2021). Bioprinting of Small-Diameter Blood Vessels. Engineering.

[22] Traore M.A., George S.C. (2017). Tissue Engineering the Vascular Tree. Tissue Eng. Part B Rev..

[23] Simunovic F., Finkenzeller G. (2021). Vascularization Strategies in Bone Tissue Engineering. Cells.

[24] Mori N., Morimoto Y., Takeuchi S. (2017). Skin integrated with perfusable vascular channels on a chip. Biomaterials.

[25] Skylar-Scott M.A., Uzel S.G.M., Nam L.L., Ahrens J.H., Truby R.L., Damaraju S., Lewis J.A. (2019). Biomanufacturing of organ-specific tissues with high cellular density and embedded vascular channels. Sci. Adv..

[26] Jia W., Gungor-Ozkerim P.S., Zhang Y.S., Yue K., Zhu K., Liu W., Pi Q., Byambaa B., Dokmeci M.R., Shin S.R. (2016). Direct 3D bioprinting of perfusable vascular constructs using a blend bioink. Biomaterials.

[27] Noor N., Shapira A., Edri R., Gal I., Wertheim L., Dvir T. (2019). 3D Printing of Personalized Thick and Perfusable Cardiac Patches and Hearts. Adv. Sci..

[28] Debbi L., Zohar B., Shuhmaher M., Shandalov Y., Goldfracht I., Levenberg S. (2020). Integrating Engineered Macro Vessels with Self-assembled Capillaries in 3D Implantable Tissue for Promoting Vascular Integration In-vivo. BioRxiv.

[29] Lee V.K., Lanzi A.M., Ngo H., Yoo S.-S., Vincent P.A., Dai G. (2014). Generation of Multi-scale Vascular Network System within 3D Hydrogel Using 3D Bio-printing Technology. Cell. Mol. Bioeng..

[30] Salameh S., Tissot N., Caché K., Lima J., Suzuki I., Marinho P.A., Rielland M., Soeur J., Takeuchi S., Germain S. (2021). A perfusable vascularized full-thickness skin model for potential topical and systemic applications. Biofabrication.

[31] Ma X., Qu X., Zhu W., Li Y.-S., Yuan S., Zhang H., Liu J., Wang P., Lai C.S.E., Zanella F. (2016). Deterministically patterned biomimetic human iPSC-derived hepatic model via rapid 3D bioprinting. Proc. Natl. Acad. Sci. USA.

[32] Zhang S.Y., Mahler G.J. (2021). Modelling Renal Filtration and Reabsorption Processes in a Human Glomerulus and Proximal Tubule Microphysiological System. Micromachines.

[33] Zheng F., Derby B., Wong J. (2021). Fabrication of microvascular constructs using high resolution electrohydrodynamic inkjet printing. Biofabrication.

[34] Campbell K.T., Stilhano R.S., Silva E.A. (2018). Enzymatically degradable alginate hydrogel systems to deliver endothelial progenitor cells for potential revasculature applications. Biomaterials.

[35] Wu P.K., Ringeisen B.R. (2010). Development of human umbilical vein endothelial cell (HUVEC) and human umbilical vein smooth muscle cell (HUVSMC) branch/stem structures on hydrogel layers via biological laser printing (BioLP). Biofabrication.

[36] Gaebel R., Ma N., Liu J., Guan J., Koch L., Klopsch C., Gruene M., Toelk A., Wang W., Mark P. (2011). Patterning human stem cells and endothelial cells with laser printing for cardiac regeneration. Biomaterials.

[37] Gruene M., Pflaum M., Hess C., Diamantouros S., Schlie S., Deiwick A., Koch L., Wilhelmi M., Jockenhoevel S., Haverich A. (2011). Laser printing of 3-D multicellular arrays for studies of cell–cell and cell–environment interactions. Tissue Eng. Part C.

[38] Kérourédan O., Bourget J.-M., Rémy M., Crauste-Manciet S., Kalisky J., Catros S., Thébaud N.B., Devillard R. (2019). Micropatterning of endothelial cells to create a capillary-like network with defined architecture by laser-assisted bioprinting. J. Mater. Sci. Mater. Med..

[39] Trujillo S., Gonzalez-Garcia C., Rico P., Reid A., Windmill J., Dalby M.J., Salmeron-Sanchez M. (2020). Engineered 3D hydrogels with full-length fibronectin that sequester and present growth factors. Biomaterials.

[40] Koch L., Deiwick A., Schlie S., Michael S., Gruene M., Coger V., Zychlinski D., Schambach A., Reimers K., Vogt P.M. (2012). Skin tissue generation by laser cell printing. Biotechnol. Bioeng..

[41] Michael S., Sorg H., Peck C.-T., Koch L., Deiwick A., Chichkov B., Vogt P.M., Reimers K. (2013). Tissue Engineered Skin Substitutes Created by Laser-Assisted Bioprinting Form Skin-Like Structures in the Dorsal Skin Fold Chamber in Mice. PLoS ONE.

[42] Gruene M., Deiwick A., Koch L., Schlie S., Unger C., Hofmann N., Bernemann I., Glasmacher B., Chichkov B. (2011). Laser Printing of Stem Cells for Biofabrication of Scaffold-Free Autologous Grafts. Tissue Eng. Part C Methods.

[43] Koch L., Deiwick A., Franke A., Schwanke K., Haverich A., Zweigerdt R., Chichkov B.N. (2018). Laser bioprinting of human induced pluripotent stem cells—The effect of printing and biomaterials on cell survival, pluripotency, and differentiation. Biofabrication.

[44] Koch L., Kuhn S., Sorg H., Gruene M., Schlie S., Gaebel R., Polchow B., Reimers K., Stoelting S., Ma N. (2010). Laser Printing of Skin Cells and Human Stem Cells. Tissue Eng. Part C Methods.

[45] Krump-Konvalinkova V., Bittinger F., E Unger R., Peters K., Lehr H.-A., Kirkpatrick C.J. (2001). Generation of human pulmonary microvascular endothelial cell lines. Lab. Investig..

[46] Unger R.E., Konvalinkova V.K.-, Peters K., Kirkpatrick C. (2002). In Vitro Expression of the Endothelial Phenotype: Comparative Study of Primary Isolated Cells and Cell Lines, Including the Novel Cell Line HPMEC-ST1.6R. Microvasc. Res..

[47] Siveen K.S., Prabhu K., Krishnankutty R., Kuttikrishnan S., Tsakou M., Alali F., Dermime S., Mohammad R.M., Uddin S. (2017). Vascular Endothelial Growth Factor (VEGF) Signaling in Tumour Vascularization: Potential and Challenges. Curr. Vasc. Pharmacol..

